# Identification and validation of an immunogenic subtype of gastric cancer with abundant intratumoural CD103^+^CD8^+^ T cells conferring favourable prognosis

**DOI:** 10.1038/s41416-020-0813-y

**Published:** 2020-03-24

**Authors:** Ruochen Li, Hao Liu, Yifan Cao, Jieti Wang, Yifan Chen, Yangyang Qi, Kunpeng Lv, Xin Liu, Kuan Yu, Chao Lin, Heng Zhang, Hongyong He, He Li, Lingli Chen, Zhenbin Shen, Jing Qin, Weijuan Zhang, Yihong Sun, Jiejie Xu

**Affiliations:** 1 Department of General Surgery, Zhongshan Hospital, Fudan University, Shanghai, China; 2 Department of Gastric Surgery, Shanghai Cancer Center, Fudan University, Shanghai, China; 3 Department of Immunology, School of Basic Medical Sciences, Fudan University, Shanghai, China; 4 Department of Biochemistry and Molecular Biology, School of Basic Medical Sciences, Fudan University, Shanghai, China; 5 Department of Pathology, Zhongshan Hospital, Fudan University, Shanghai, China

**Keywords:** Cancer microenvironment, Immunoediting, Gastric cancer

## Abstract

**Background:**

Intratumoural CD103^+^CD8^+^ T cells have been linked to prolonged survival in several malignancies. However, the clinical significance of CD103^+^CD8^+^ T cells in gastric cancer remains unexplored.

**Methods:**

Gastric cancer tissues from Zhongshan Hospital and data from Gene Expression Omnibus were obtained and analysed. Immunohistochemistry and flow cytometry were performed to detect the number and phenotypical characteristics of CD103^+^CD8^+^ T cells. The effect of programmed cell death protein-1 (PD-1) blockade on CD103^+^CD8^+^ T cells was evaluated with the use of an in vitro study based on fresh tumour tissues.

**Results:**

CD103^+^CD8^+^ T cells predicted superior overall survival and provided better prognostic power than total CD8^+^ T cells in gastric cancer. Patients with high CD103^+^CD8^+^ T cell infiltration also gained more benefit from adjuvant chemotherapy. Flow cytometry analysis showed that CD103^+^CD8^+^ T cells exerted superior anti-tumour effects with stronger retention capacity and cytotoxicity. Moreover, an in vitro study showed that CD103^+^CD8^+^ T cells were more functionally restored after PD-1 blockade than CD103^-^CD8^+^ T cells.

**Conclusions:**

CD103^+^CD8^+^ T cells might be a useful marker to predict prognosis and therapeutic efficacy for gastric cancer patients. Efforts to increase intratumoural CD103^+^CD8^+^ T cell frequency might be a novel therapeutic strategy in gastric cancer.

## Background

Gastric cancer is the fifth most common cancer and the third leading cause of cancer-related death worldwide.^1^ In recent years, although significant progress has been made in the prevention, diagnosis and therapeutic strategies of gastric cancer, many questions remain unanswered, especially the prediction of prognosis and therapeutic response in gastric cancer.^2^ Currently, it is generally believed that the pathogenies and progression of gastric cancer are influenced by the cross-talk between tumour cells and the host immune system.^3–5^ Consequently, the prognostic and predictive value of tumour-infiltrating immune cells in gastric cancer has drawn more attention in the past ten years.^6–8^

CD8^+^ T cells play a central role in anti-tumour immunity, and increased CD8^+^ T cell infiltration usually indicates better prognosis in solid cancers.^9–11^ However, the prognostic value of CD8^+^ T cell infiltration is still controversial in gastric cancer.^12,13^ In fact, the CD8^+^ T cell compartment in tumour tissues is largely diverse, comprising several subsets with different degrees of specialisation in phenotype, function, and gene expression.^14^ Therefore, to understand the prognostic implication of tumour-infiltrating CD8^+^ T cells and to identify valuable predictive biomarkers for therapeutic response, further classification of CD8^+^ T cell subsets based on phenotypic and functional characteristics is urgently needed.

CD103, also known as integrin αEβ7 (ITGAE), is a transmembrane heterodimer complex that mediates cell adhesion, migration and homing of lymphocytes through binding to E-cadherin on the surface of epithelial cells.^15^ Recently, several studies have reported that CD103^+^CD8^+^ T cells might represent a subset of activated tumour-reactive CD8^+^ T cells and predict better prognosis in a series of malignancies.^16–18^ However, the clinical significance and precise phenotypic features of intratumoural CD103^+^CD8^+^ T cells in gastric cancer have never been reported before. Consequently, our current study was designed to evaluate the prognostic value and to explore the phenotypic characteristics of intratumoural CD103^+^CD8^+^ T cells in gastric cancer.

Here, we found that intratumoural CD103^+^CD8^+^ T cell infiltration was a stronger prognostic factor than total CD8^+^ T cell infiltration in gastric cancer. Phenotypic analysis showed that CD103^+^CD8^+^ T cells exhibited tissue-resident features and higher cytotoxic activity than total CD103^-^CD8^+^ T cells. Moreover, CD103^+^CD8^+^ T cells expressed higher levels of coinhibitory receptors than CD103^-^CD8^+^ T cells and had the potential to be target cells for immunotherapy in gastric cancer. Conclusively, our results suggested that CD103^+^CD8^+^ T cells played an important role in anti-tumour immunity and could be a useful prognostic and predictive biomarker in gastric cancer.

## Methods

### Study population

Initially, data from 496 gastric cancer patients who underwent radical gastrectomy between 2007 and 2008 were obtained from Zhongshan Hospital. However, only 468 of the 496 patients had comprehensive information about chemotherapy, clinicopathological data and survival outcomes for complete analysis. In this study, nine patients with distant metastasis were excluded, and 11 dots on the tissue microarrays (TMAs) were lost after immunohistochemistry. Consequently, we included 448 patients from Zhongshan Hospital (Zhongshan Cohort) in our study. Demographic and clinical data were collected retrospectively. Cancer stages were determined according to the 7th edition of the American Joint Committee on Cancer (AJCC) TNM classification. Postoperative adjuvant chemotherapy (ACT) was administered to patients according to NCCN guidelines for gastric cancer and patients’ wills. Two independent public datasets, GSE84437 and GSE62254, were employed for external validation. After excluding patients with distant metastasis or incomplete data, 431 patients from GSE84437 and 220 patients from GSE62254 were included in the subsequent analysis. Additionally, a total of 36 fresh tumour tissue samples were obtained during surgery at the Department of General Surgery of Zhongshan Hospital for flow cytometry analysis. Written informed consent was obtained from each patient, and the study was approved by the institutional review board and ethics committee of Zhongshan Hospital, Fudan University.

### Multicolour immunohistochemistry and immunofluorescence analysis

The TMAs of the Zhongshan cohort were constructed as previously described.^19^ Dual immunohistochemistry (IHC) staining was performed. Briefly, the TMA slides were dewaxed in an oven and treated with water bath-heated xylene and graded alcohols. The slides were heated in sodium citrate buffer (0.01 M sodium citrate buffer, pH = 6) for 15 min for antigen retrieval. Normal goat serum blocking solution was applied for 20 min at 37 °C. Then, anti-CD103 antibody (Abcam, ab129202, diluted at 1:300) was applied for 2 h at 37 °C. After the primary antibody incubation, a general two-step kit detection system was used (HRP, Mo/Ra; ZSGB Biotech, PV-9000) with DAB. The slides were washed again and incubated with anti-CD8A primary antibody (Abcam, ab199016, diluted at 1:1500) overnight at 4 °C. TMA slides were subsequently washed, incubated with AP-labelled secondary antibody and stained with Vector Blue (Vector Blue AP Substrate Kit detection system; Vector Labs, SK-5300). Finally, the sections were washed, dehydrated and mounted. For immunofluorescence staining, the sections were incubated with the primary antibodies overnight at 4 °C. Then, samples were incubated with FITC-conjugated and TRITC-conjugated secondary antibodies for 2 h at 37 °C. Finally, the slides were mounted with anti-fade mounting solution containing DAPI. The slides were captured using a Leica DMi8 microscope.

### Evaluation of the CD103^+^CD8^+^ T cell density in IHC specimens

**Fig. 1:**
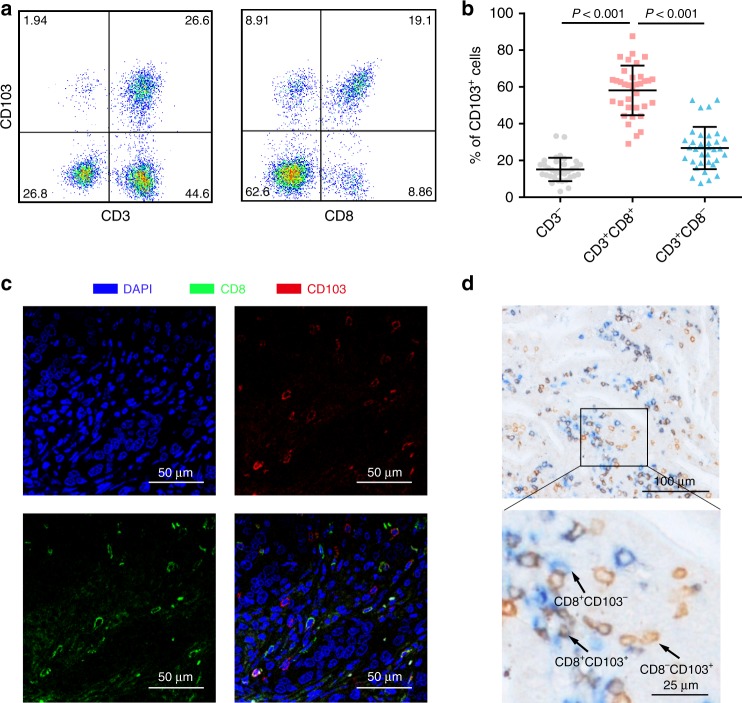
Identification of CD103^+^CD8^+^ T cells in human gastric cancer tissues. **a** Representative images from flow cytometry showing the co-expression of CD103 with the T cell surface markers CD3 and CD8 (gated on the CD45^+^ subset). **b** Scatter plot showing the fractions of CD103^+^ immune cells in gastric cancer (*n* = 36). **c** Representative immunofluorescence staining of CD8 and CD103 in gastric cancer tissues. **d** Representative dual immunohistochemical staining of CD8 and CD103 in gastric cancer tissues. Bar plots show the mean ± SD.

After dual IHC staining, CD103 and CD8 were stained brown and blue, respectively. CD103^+^CD8^+^ T cells were dark brown and could be easily distinguished from single-positive cells (Fig. 1d). The three most representative high-power fields were captured at ×200 magnification (0.284 mm^2^ per field) for each tumour region in all specimens under a Nikon eclipse Ti-s microscope (Nikon, Tokyo, Japan). Then, two pathologists (L. Chen and P. Zhang) who were blinded to the patients’ clinical data determined the number of CD103^+^CD8^+^ cells (cells stained in dark brown), CD103^-^CD8^+^ cells (cells stained in blue) and CD103^+^CD8^-^ cells (cells stained in light brown) in each field. Additionally, the numbers of CD103^+^CD8^+^ and CD103^-^CD8^+^ cells were summed as the number of total CD8^+^ T cells, while the number of total CD103^+^ cells was the sum of CD103^+^CD8^+^ and CD103^+^CD8^-^ cells. In case of disagreement, the pictures were reviewed, and a consensus was reached by the two observers. Finally, three fields were averaged as the ultimate score of each sample.

### Flow cytometry

Freshly isolated gastric cancer tissues were prepared as previously reported.^20^ Briefly, after the red blood cells were lysed, samples were incubated with Human BD Fc Block (BD Biosciences) and then stained with the indicated monoclonal antibodies (mAbs) for 30 min at 4 °C in the dark. If necessary, the Fixation/Permeabilization Solution Kit and Transcription Factor Fixation/Permeabilization buffer set (BD Biosciences) were used according to the manufacturer’s instructions. Stained cells were washed and resuspended in cell staining buffer. Cell apoptosis was assessed with the use of FITC Annexin V Apoptosis Detection Kit I following the manufacturer’s instructions. Subsequently, the stained cells were separated with a FACSCelesta flow cytometer (BD Biosciences) and analysed with FlowJo software (Tree Star). The following human antibodies (clones) were used: anti-CD45 (2D1), anti-CD3 (HIT3a), anti-CD8 (RPA-T8), anti-CD49a (TS2/7), anti-CCR7 (3D12), anti-TCF-1 (7F11A10), anti-granzyme-B (QA16A02), anti-CTLA-4 (BNI3), anti-LAG-3 (11C3C65), anti-IFN-γ (4 S. B3), anti-TNF-α (MAb11), anti-CD326 (Epcam, 8C4) and anti-Ki-67 (Cat#350522, clone number not provided) were from BioLegend; anti-CD103 (Ber-ACT8), anti-CD69 (FN50), anti-RUNX3 (R3-5G4), anti-BLIMP-1 (6D3), anti-HOBIT (Sanquin-Hobit/1), anti-CD107a (H4A3), anti-perforin (δG9), anti-TIM-3 (7D3), anti-IL-2 (MQ1-17H12) and FITC Annexin V Apoptosis Detection Kit I were from BD Biosciences; anti-CD62L (LT-TD180) and anti-PD-1 (MIH4) were from eBioscience. The detailed experimental setups are shown in Table S1. Flow cytometry results were analysed with FlowJo software (Tree Star).

### In vitro therapeutic assay

Tumour single-cell suspensions were obtained from fresh tumour tissues as mentioned above. The digests were cultured in assay medium (RPMI-1640 medium with 100 U/mL penicillin, 100 µg/mL streptomycin and 10% foetal bovine serum) with anti-CD3 antibody (0.5 µg/ml, OKT3; BioLegend) and anti-CD28 antibody (2 μg/ml, clone 28.2; eBioscience) in the presence of pembrolizumab (5 µg/ml; Selleck) or isotype control as previously described.^21^ After 24 h of incubation, cells were collected, washed, and analysed by flow cytometry.

### Public dataset analysis

The GEO datasets GSE84437 and GSE62254 were downloaded from the respective repository in the Gene Expression Omnibus (https://www.ncbi.nlm.nih.gov/geo/). Intratumoural CD8^+^ T cell abundance was estimated based on microarray data by the CIBERSORT algorithm using LM22 as the expression signature.^22^ As CIBERSORT did not contain the CD103^+^CD8^+^ T cell subset, we used the single sample gene set enrichment analysis (ssGSEA) programme to estimate the relative abundance of intratumoural CD103^+^CD8^+^ T cells. In brief, we first obtained a marker geneset of CD103^+^CD8^+^ T cells from MSigDB (GSE39152_CD103_NEG_VS_POS_MEMORY_CD8_TCELL_DN). Next, the gene set variation analysis (GSVA) R package and its ssGSEA method (http://www.bioconductor.org) were implemented to further obtain the GSVA scores of the marker gene set for each sample in the two GEO datasets.^23,24^ The GSVA score represented the degree of absolute enrichment of the CD103^+^CD8^+^ T cell marker geneset in each sample, which indicated the relative abundance of intratumoural CD103^+^CD8^+^ T cells.

### Statistical analysis

Statistical analysis was performed using GraphPad Prism (version 6.00), R software (version 3.5.3), MedCalc (version 12.7.0) or IBM SPSS Statistics (version 21) software. The cut-off values to discriminate high- and low-density groups were determined by the minimum P method using X-tile software for all markers in all three independent cohorts. Ultimately, 37/HPF, -0.007593 and 0.268 were determined as the cut-off values for CD103^+^CD8^+^ T cells in the Zhongshan cohort, GSE84437 and GSE62254, respectively. The survival outcomes between two patient subpopulations were compared using the Kaplan–Meier method and log-rank test. Comparisons between two sample groups were made with Student’s *t*-test. Correlations between the data were assessed using Spearman’s correlation test. Grouped data in the figures are represented by the means ± SDs. For all tests in this study, a *P*-value of <0.05 was considered statistically significant.

## Results

### The presence of CD103^+^CD8^+^ T cells in gastric cancer and its association with clinicopathological features

Flow cytometric analysis of single-cell suspensions of human gastric cancer tissues revealed that CD103 was predominantly expressed on CD3^+^CD8^+^ T cells in gastric cancer tissues (Fig. 1a, b). Then, we confirmed the co-expression of CD103 and CD8 on tumour-infiltrating lymphocytes in formalin-fixed paraffin-embedded gastric cancer tissues by immunofluorescence (Fig. 1c). To assess the density of intratumoural CD103^+^CD8^+^ T cells in gastric cancer tissue microarrays, double staining immunohistochemistry was employed (Fig. 1d). Afterwards, we analysed the association between CD103^+^CD8^+^ T cell infiltration and clinicopathologic features in the three independent cohorts, but no significant association was found (Table S2).

### CD103^+^CD8^+^ T cells have superior prognostic ability in patients with gastric cancer

**Fig. 2:**
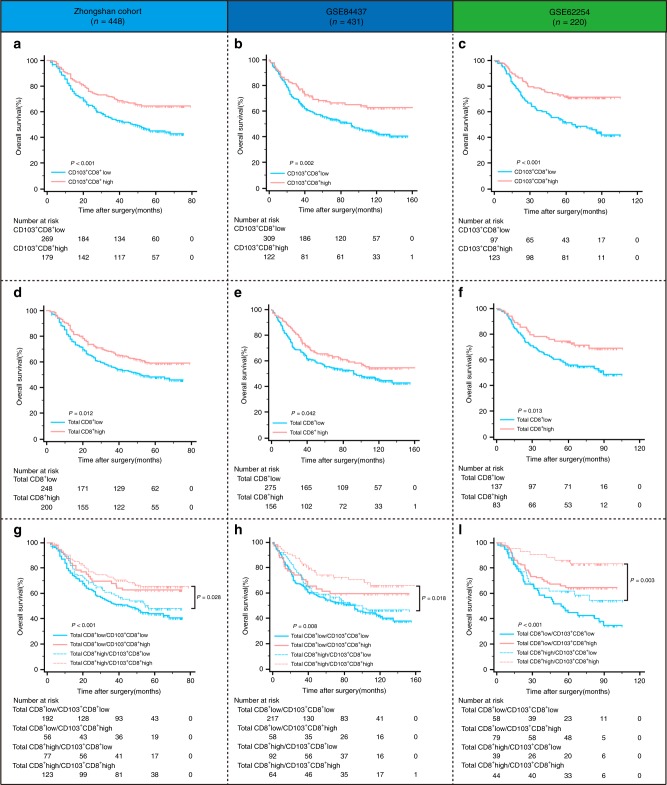
The prognostic value of intratumoural CD103^+^CD8^+^ T cells in patients with gastric cancer. **a**–**c** Kaplan–Meier survival curves for overall survival of gastric cancer patients from the Zhongshan cohort (**a**), GSE84437 (**b**) and GSE62254 (**c**) according to intratumoural CD103^+^CD8^+^ T cell infiltration. **d**–**f** Kaplan–Meier survival curves for overall survival of gastric cancer patients from the Zhongshan cohort (**d**), GSE84437 (**e**) and GSE62254 (**f**) according to total CD8^+^ T cell infiltration. **g**–**i** Kaplan–Meier survival analysis for gastric cancer patients further stratified according to CD103^+^CD8^+^ T cells within total CD8^+^ T cell strata.

To evaluate the clinical significance of CD103^+^CD8^+^ T cells in anti-tumour immunity, Kaplan–Meier analysis was performed in the three independent cohorts of gastric cancer. A higher density of intratumoural CD103^+^CD8^+^ T cells was associated with improved overall survival (OS) in all three cohorts (Fig. 2a, b, c; *P* < 0.001, *P* = 0.002 and *P* < 0.001, respectively). Although CD8^+^ T cells were associated with patient survival, it was probable that some intratumoural CD8^+^ T cells were only bystanders rather than active participants in the immune response. Consequently, we intended to explore whether CD103 expression on intratumoural CD8^+^ T cells could identify a CD8^+^ T cell subset with superior prognostic power in gastric cancer. A higher density of total CD8^+^ T cells indicated improved overall survival in the three cohorts (Fig. 2d, e, f; *P* = 0.012, *P* = 0.042 and *P* = 0.013, respectively). Interestingly, when we combined CD8^+^ T cells with CD103^+^CD8^+^ T cells for survival analysis, we found that CD103^+^CD8^+^ T cell density could further stratify the patients with high CD8^+^ T cell infiltration into two subgroups with significant survival differences (Fig. 2g, h, i; *P* = 0.028, *P* = 0.018 and *P* = 0.003, respectively). These results suggested that CD103^+^CD8^+^ T cells had superior prognostic abilities compared with total CD8^+^ T cells in gastric cancer.

### Increased CD103^+^CD8^+^ T cell infiltration predicts better efficacy of adjuvant chemotherapy after surgery in stage II/III gastric cancer patients

**Fig. 3:**
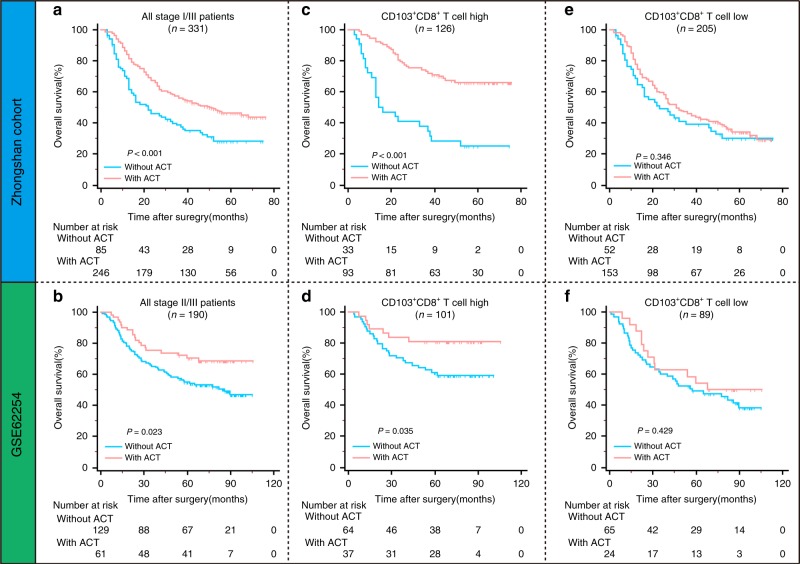
Predictive value of intratumoural CD103^+^CD8^+^ T cells in response to adjuvant chemotherapy for gastric cancer patients. **a**, **b** Kaplan–Meier survival curves for overall survival of all stage II/III patients from the Zhongshan cohort (**a**) and GSE62254 (**b**) according to ACT treatment. **c**, **d** Kaplan-Meier survival curves for overall survival of patients with high CD103^+^CD8^+^ T cell infiltration from the Zhongshan cohort (**c**) and GSE62254 (**d**) according to ACT treatment. **e**, **f** Kaplan-Meier survival curves for overall survival of patients with low CD103^+^CD8^+^ T cell infiltration from the Zhongshan cohort (**e**) and GSE62254 (**f**) according to ACT treatment.

In the Zhongshan cohort and GSE62254, treatment with fluorouracil-based ACT was associated with better OS in patients with TNM II/III gastric cancer (Fig. 3a, b), a relationship that was identified by the CLASSIC trials.^25^ To evaluate the predictive value of intratumoural CD103^+^CD8^+^ T cells for ACT, we investigated the association between ACT and OS among patients with TNM II/III stage disease in the Zhongshan cohort and GSE62254 belonging to different CD103^+^CD8^+^ T cell infiltration groups. For patients with high CD103^+^CD8^+^ T cell infiltration, ACT provided a significant survival benefit in both the Zhongshan cohort and GSE62254 (*P* < 0.001 and *P* = 0.035, respectively; Fig. 3c, d). Interestingly, ACT did not improve the overall survival of patients with low CD103^+^CD8^+^ T cell infiltration in the two cohorts (*P* = 0.346 and *P* = 0.429, respectively; Fig. 3e, f). These results indicated that intratumoural CD103^+^CD8^+^ T cell infiltration could identify patients who might benefit more from adjuvant chemotherapy.

### Intratumoural CD103^+^CD8^+^ T cells show tissue residency features

**Fig. 4:**
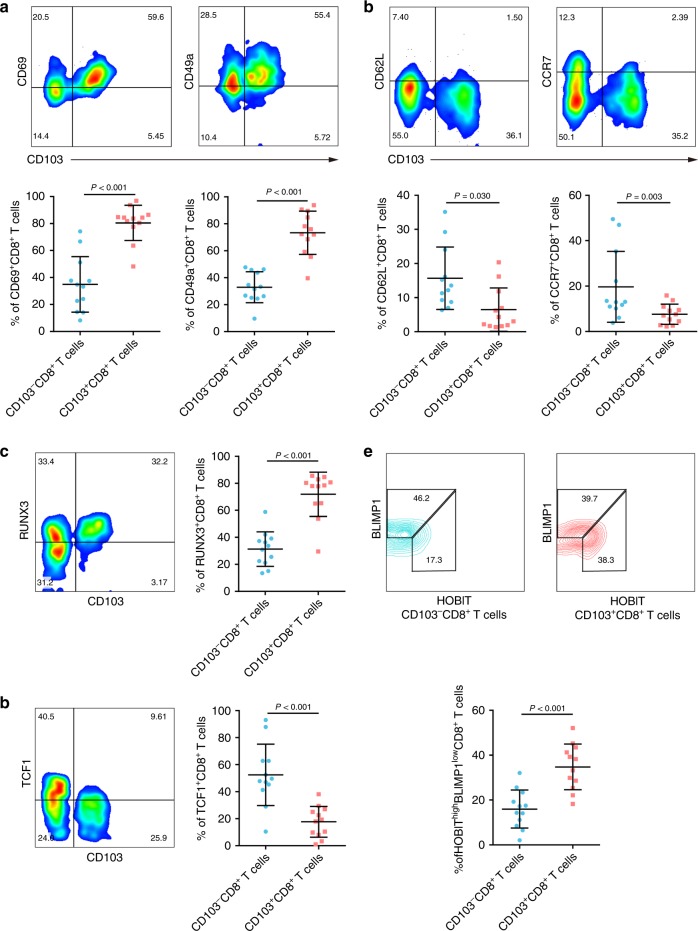
Intratumoural CD103^+^CD8^+^ T cells display features of tissue residency. **a** Flow cytometry analysis of tissue-retention markers (CD69 and CD49a) in CD103^-^CD8^+^ and CD103^+^CD8^+^ T cells from gastric cancer tissues (*n* = 12). **b** Flow cytometry analysis of tissue-egression markers (CCR7 and CD62L) in CD103^-^CD8^+^ and CD103^+^CD8^+^ T cells from gastric cancer tissues (*n* = 12). **c** Flow cytometry analysis of the transcription factor RUNX3 in CD103^-^CD8^+^ and CD103^+^CD8^+^ T cells from gastric cancer tissues (*n* = 12). **d** Flow cytometry analysis of the transcription factor TCF-1 in CD103^-^CD8^+^ T cells and CD103^+^CD8^+^ T cells from gastric cancer tissues (*n* = 12). **e** Expression levels of the transcription factor BLIMP-1 and its homologue HOBIT in CD103^-^CD8^+^ and CD103^+^CD8^+^ T cells from gastric cancer tissues (*n* = 12). Bar plots show the mean ± SD; significance was assessed by a paired *t*-test. n.s. not significant.

Previous studies have indicated that CD103^+^CD8^+^ T cells are a subset that mainly reside in non-lymphoid tissues,^26^ so we explored whether they retain this characteristic in gastric cancer. By flow cytometry, we found that CD103^+^CD8^+^ T cells expressed higher levels of molecules involved in tissue retention of lymphocytes, such as CD69 and CD49a, than their counterparts (Fig. 4a). In contrast, CCR7 and CD62L, which are associated with tissue egress, were rarely expressed by the CD103^+^CD8^+^ T cell subset (Fig. 4b). Given the distinct expression of tissue retention- and tissue egress-related molecules, we assumed that this tissue residency paradigm was regulated by a unique repertoire of transcription factors. In our following study, a higher percentage of RUNX3^+^ and HOBIT^high^/BLIMP-1^low^ cells was observed in the CD103^+^CD8^+^ T cell than in the CD103^-^CD8^+^ T cells (Fig. 4c, e). However, TCF1, a transcriptional activator of the genes encoding CCR7 and CD62L, showed a significantly lower frequency in the CD103^+^CD8^+^ T cell subset than in CD103^+^CD8^+^ T cells (Fig. 4d). Conclusively, these results showed that intratumoural CD103^+^CD8^+^ T cells displayed tissue residency features.

### Intratumoural CD103^+^CD8^+^ T cells exhibit a highly activated phenotype in gastric cancer

**Fig. 5:**
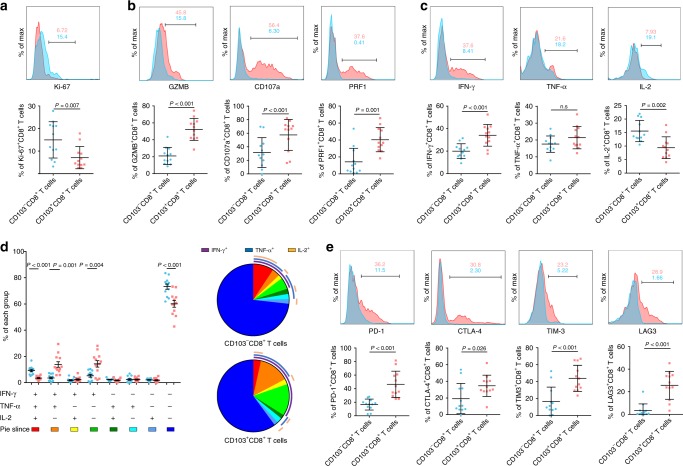
Functional characteristics of intratumoural CD103^+^CD8^+^ T cells in gastric cancer tissues. **a** Flow cytometry analysis of the proliferation marker Ki-67 in CD103^-^CD8^+^ and CD103^+^CD8^+^ T cells from gastric cancer tissues (*n* = 12). **b** Flow cytometry analysis of cytolytic markers (GZMB, CD107a and PRF1) in CD103^-^CD8^+^ and CD103^+^CD8^+^ T cells from gastric cancer tissues (*n* = 12). **c** Flow cytometry analysis of effector cytokines (IFN-γ, TNF-α and IL-2) in CD103^-^CD8^+^ and CD103^+^CD8^+^ T cells from gastric cancer tissues (*n* = 12). **d** Frequency of subsets with different combinations of IFN-γ, TNF-α and IL-2 in CD103^-^CD8^+^ and CD103^+^CD8^+^ T cells from gastric cancer tissues (*n* = 12) (left panel). Summary pie graphs representing the proportions of subsets with different combinations of IFN-γ, TNF-α and IL-2 in CD103^-^CD8^+^ and CD103^+^CD8^+^ T cells from gastric cancer tissues (right panel). **e** Flow cytometry analysis of coinhibitory receptors (PD-1, CTLA-4, TIM-3 and LAG-3) in CD103^-^CD8^+^ and CD103^+^CD8^+^ T cells from gastric cancer tissues (*n* = 12). GZMB granzyme B, IFN-γ interferon gamma, PRF1 perforin, IL-2 interleukin-2, PD-1 programmed cell death protein-1, CTLA-4 cytotoxic T lymphocyte-associated protein 4, TIM-3 T cell immunoglobulin domain and mucin domain 3, LAG-3 lymphocyte activation gene 3. Bar plots show the mean ± SD. Significance was assessed by paired *t*-test. n.s. not significant.

We investigated the unique functional phenotype of intratumoural CD103^+^CD8^+^ T cells. A lower frequency of Ki-67^+^ cells was found in CD103^+^CD8^+^ T cells than in CD103^−^CD8^+^ T cells, which indicated that CD103^+^CD8^+^ cells had a lower proliferative ability. However, effector molecules related to cytolytic activity, such as granzyme B (GZMB), perforin (PRF1) and CD107a, were highly expressed by CD103^+^CD8^+^ T cells. The effector function of CD8^+^ T cells was also assessed by analysing their ability to produce cytokines, including interleukin-2 (IL-2), interferon-gamma (IFN-γ) and tumour necrosis factor-alpha (TNF-α). Interestingly, CD103^+^CD8^+^ T cells showed only higher IFN-γ expression (Fig. 5c). Furthermore, we examined the co-expression of the three cytokines (IFN-γ, IL-2 and TNF-α) and found an increased frequency of IFN-γ^+^TNF-α^+^IL-2^−^ and IFN-γ^+^TNF-α^−^IL-2^−^ cells but a decreased frequency of IFN-γ^−^TNF-α^−^IL-2^+^ cells among CD103^+^CD8^+^ T cells compared with the respective numbers in their CD103^−^ counterparts (Fig. 5d). Notably, CD103^+^CD8^+^ T cells also expressed higher levels of coinhibitory receptors in gastric cancer than CD103^−^CD8^+^ T cells (Fig. 5e). Collectively, these data showed that intratumoural CD103^+^CD8^+^ T cells displayed a highly activated phenotype with lower proliferative potential, higher cytotoxicity and higher coinhibitory receptor expression than CD103^−^CD8^+^ T cells, as well as a distinct cytokine production capacity.

### Intratumoural CD103^+^CD8^+^ T cells are more functionally restored following PD-1 blockade in gastric cancer than CD103^−^CD8^+^ T cells

**Fig. 6:**
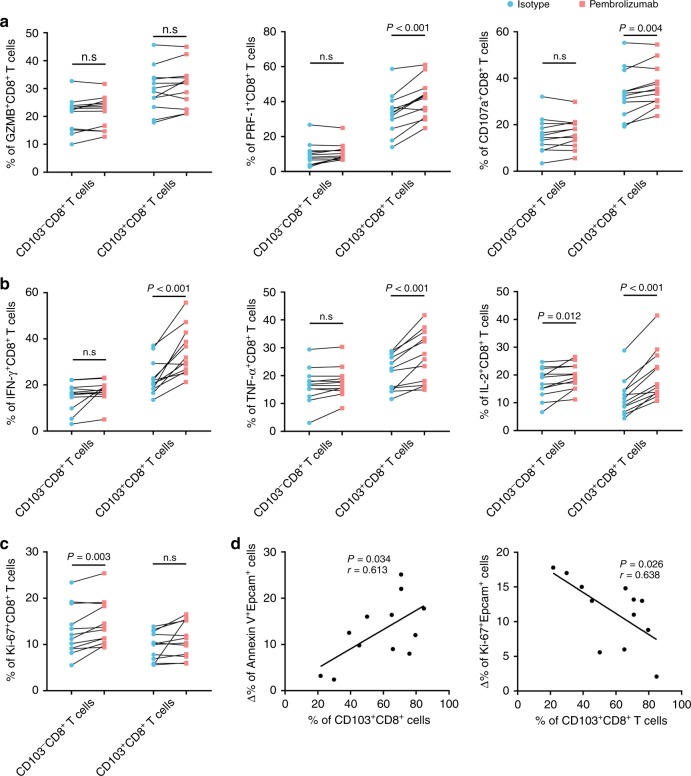
Intratumoural CD103^+^CD8^+^ T cells are functionally restored following PD-1 blockade. **a** The effect of pembrolizumab on the expression of cytolytic markers (GZMB, CD107a and PRF1) in CD103^-^CD8^+^ T cells and CD103^-^CD8^+^ T cells from gastric cancer tissue samples (*n* = 12). **b** The effect of pembrolizumab on the expression of effector cytokines (IFN-γ, TNF-α and IL-2) in CD103^-^CD8^+^ T cells and CD103^-^CD8^+^ T cells from gastric cancer tissue samples (*n* = 12). **c** The effect of pembrolizumab on the proliferation capacity of CD103^-^CD8^+^ T cells and CD103^-^CD8^+^ T cells from gastric cancer tissue samples (*n* = 12). **d** Association of CD103^+^CD8^+^ T cell frequencies with the change in apoptotic (left panel) and proliferative (right panel) tumour cells induced by pembrolizumab in gastric cancer tissue samples (*n* = 12). Bar plots show the mean ± SD; GZMB granzyme B, IFN-γ interferon gamma, PRF1 perforin, IL-2 interleukin-2. Bar plots show the mean ± SD. Significance was assessed by paired *t*-test. n.s. not significant.

Previous studies have shown that CD103^+^CD8^+^ T cells rather than CD103^−^CD8^+^ T cells are promising targets for immune checkpoint inhibitors.^27^ Consequently, we further examined whether CD103^+^CD8^+^ T cells and CD103^−^CD8^+^ T cells respond differently to PD-1 blockade in gastric cancer. Flow cytometry analysis showed that PD-1 blockade had a more profound effect on the effector molecule expression of CD103^+^CD8^+^ T cells than on that of CD103^−^CD8^+^ T cells (Fig. 6a, b). However, we found that CD103^−^CD8^+^ T cells, rather than CD103^+^CD8^+^ T cells, showed a proliferation burst after PD-1 blockade (Fig. 6c). Notably, there were more Annexin V^+^ epithelial cells and fewer Ki-67^+^ epithelial cells with the increase in intratumoural CD103^+^CD8^+^ T cell frequency after pembrolizumab treatment (Fig. 6d). These data suggested that CD103^+^CD8^+^ T cells were more responsive to PD-1 blockade than CD103^−^CD8^+^ T cells and had the potential to be a predictor for immunotherapeutic efficacy.

## Discussion

In this study, we characterised the prognostic value, phenotype and function of intratumoural CD103^+^CD8^+^ T cells in gastric cancer. We found that CD103^+^CD8^+^ T cell infiltration had strong prognostic value and indicated a superior response to adjuvant chemotherapy in gastric cancer. Intratumoural CD103^+^CD8^+^ T cells retained tissue-resident properties in gastric cancer, as do those in other non-lymphoid tissues. Flow cytometry analysis showed that CD103^+^CD8^+^ T cells had stronger cytotoxic capacity as well as coinhibitory receptor expression than CD103^−^CD8^+^ T cells. Moreover, we found that CD103^+^CD8^+^ T cells were more effectively reinvigorated by PD-1 blockade than CD103^−^CD8^+^ T cells. These findings highlight the critical role of CD103^+^CD8^+^ T cells in the immune response against gastric cancer.

Prognostic assessment is crucial for appropriate treatment choices. Recently, the prognostic significance of tumour-infiltrating immune cells has drawn increasing attention because of their critical role in tumorigenesis and progression. In this study, we found that patients with high infiltration of CD103^+^CD8^+^ T cells had improved overall survival in a large TMA cohort, which was further validated in two external public datasets, GSE62254 and GSE84437. To the best of our knowledge, our study is the first to identify intratumoural CD103^+^CD8^+^ T cell density as a useful prognostic factor in gastric cancer. Remarkably, our study also revealed the predictive value of intratumoural CD103^+^CD8^+^ T cells in response to ACT. For stage II/III patients, fluorouracil-based ACT is recommended as a first-line adjuvant therapy regimen.^25,28,29^ However, not everyone benefits from adjuvant chemotherapy, and the criterion for the selection of candidates is still controversial in clinical practice.^30^ To avoid excessive toxicities, it is important to identify patients who will be more sensitive to chemotherapy. Our findings will be useful for better selection and management of patients who are strongly recommended for adjuvant chemotherapy.

Considering the superior prognostic power of intratumoural CD103^+^CD8^+^ T cells compared with total CD8^+^ T cells, we further explored the phenotypic characteristics of intratumoural CD103^+^CD8^+^ T cells. CD103^+^CD8^+^ T cells in gastric cancers exhibited a similar phenotype to those in other non-lymphoid tissues, including downregulation of lymph node homing-associated molecules (e.g. CD62L, CCR7 and TCF-1)^31–33^ and upregulation of tissue residency-promoting molecules (e.g. CD69, CD49a and RUNX3),^34–36^ which indicated the tissue-resident features of CD103^+^CD8^+^ T cells in gastric cancer. Notable in this repertoire was the expression of the transcription factor HOBIT and its homologue BLIMP-1, which co-regulate the formation and/or maintenance of tissue-resident T cells.^37^ In our current study, we found that intratumoural CD103^+^CD8^+^ T cells in gastric cancer exhibited a HOBIT^high^/BLIMP-1^low^ phenotype, which was inconsistent with findings in human lung tissues.^38^ Indeed, despite their commonalities, tissue-resident lymphocytes may display a vast number of specific transcripts in different tissues or at different stages.^39^ These findings might reflect differences in signals from the local microenvironment modulating CD103^+^CD8^+^ formation.

Our study indicated that CD103^+^CD8^+^ T cells showed high levels of cytolytic enzyme expression and IFN-γ production despite the high expression of multiple inhibitory receptors. These results were consistent with O’Brien SM’s findings, which showed that CD103^+^CD8^+^ T cells produced more IFN-γ than their CD103^-^ counterparts despite high expression of coinhibitory receptors.^40^ Although a hallmark of dysfunctional T cells within the tumour microenvironment is the overexpression of coinhibitory receptors, the actual contribution of these coinhibitory receptors to the dysfunctional state is still controversial.^41–43^ Multiple studies have revealed that coinhibitory receptors are also induced when T cells are highly activated. Conclusively, we speculated that CD103^+^CD8^+^ T cells represented a highly activated T cell subset.

Interestingly, although CD103^+^CD8^+^ T cells seemed to be hyperfunctional, PD-1 blockade had a more significant influence on CD103^+^CD8^+^ T cells than their counterparts in gastric cancer. Previous studies have demonstrated that CD103^+^CD8^+^ T cells might be a major cellular target of anti-PD-1 therapy in patients with melanoma and lung cancer.^44,45^ Our data strongly suggested that CD103^+^CD8^+^ T cell density could predict the effectiveness of checkpoint blockade in vitro. Nevertheless, these findings still need further confirmation in larger, multicentre patient cohorts.

The main limitation of our study was the lack of in vivo validation because no appropriate mouse-derived cell line for gastric cancer could be acquired for building an immune-competent mouse model. Moreover, we did not identify the exact cytokines that determined the formation and differentiation of the CD103^+^CD8^+^ T cell subset in gastric cancer, and these ideas require further elucidation in our following studies. Moreover, since the minimum P method was used to determine the cut-off values, these cut-off values might not be reliable or reproducible.

In summary, we identified and validated a subtype of gastric cancer with high CD103^+^CD8^+^ T cell density that indicated superior survival outcomes and response to adjuvant chemotherapy. Further analysis showed that CD103^+^CD8^+^ T cells could possibly exert superior anti-tumour effects with stronger retention capacity and cytotoxicity than CD103^-^CD8^+^ T cells. Furthermore, CD103^+^CD8^+^ T cells are further functionally restored after PD-1 blockade, indicating that CD103^+^CD8^+^ T cells might be a potential cellular target of anti-PD-1 therapy.

## Supplementary Information

Supplementary Table S1

Supplementary Table S2

## Data Availability

All data generated that are relevant to the results presented in this article are included in this article. Other data that were not relevant for the results presented here are available from the corresponding author Dr Xu upon reasonable request.
