# Supplementary material for: Identification and validation of an immunogenic subtype of gastric cancer with abundant intratumoural CD103+CD8+ T cells conferring favourable prognosis
Source: Br J Cancer. 2020 Mar 24;122(10):1525–34. doi: 10.1038/s41416-020-0813-y (PMC7217759; doi:10.1038/s41416-020-0813-y)
Supplement: Supplementary file 1 — Supplementary Table S1 [file 41416_2020_813_MOESM1_ESM.doc]

| **Table S1. Flow cytometry experimental setups** | | | | | | | | |
| --- | --- | --- | --- | --- | --- | --- | --- | --- |
| **Panel A, n=12**  **(for detection of CD103^+^CD8^+^ T cell proportion)** | **Panel B, n=12**  **(for detection of tissue resident features)** | | | | **Panel C, n=12**  **(for detection of functional status)** | | | |
| **Tube 1** | **Tube 1** | **Tube 2** | **Tube 3** | **Tube 4** | **Tube 1** | **Tube 2** | **Tube 3** | **Tube 4** |
| PE-Cy7-CD45  (2D1,) | PE-Cy7-CD45  (2D1) | PE-Cy7-CD45  (2D1) | PE-Cy7-CD45  (2D1) | PE-Cy7-CD45  (2D1) | PE-Cy7-CD45  (2D1) | PE-Cy7-CD45  (2D1) | PE-Cy7-CD45  (2D1) | PE-Cy7-CD45  (2D1) |
| Percp-Cy5.5-CD3  (HIT3a) | Percp-Cy5.5-CD3  (HIT3a) | Percp-Cy5.5-CD3  (HIT3a) | Percp-Cy5.5-CD3  (HIT3a) | Percp-Cy5.5-CD3 (HIT3a) | Percp-Cy5.5-CD3  (HIT3a) | Percp-Cy5.5-CD3  (HIT3a) | Percp-Cy5.5-CD3  (HIT3a) | Percp-Cy5.5-CD3  (HIT3a) |
| FITC-CD8  (RPA-T8) | FITC-CD8  (RPA-T8) | FITC-CD8  (RPA-T8) | FITC-CD8  (RPA-T8) | FITC-CD8  (RPA-T8) | FITC-CD8  (RPA-T8) | FITC-CD8  (RPA-T8) | FITC-CD8  (RPA-T8) | FITC-CD8  (RPA-T8) |
| BV421-CD103  (Ber-ACT8) | BV421-CD103  (Ber-ACT8) | BV421-CD103  (Ber-ACT8) | BV421-CD103  (Ber-ACT8) | BV421-CD103  (Ber-ACT8) | BV421-CD103  (Ber-ACT8) | BV421-CD103  (Ber-ACT8) | BV421-CD103  (Ber-ACT8) | BV421-CD103  (Ber-ACT8) |
| - | PE-CCR7  (3D12) | PE-CD69  (FN50) | PE-BLIMP-1  (6D3) | PE-RUNX-3  (6D3) | PE-IFN-γ(4S.B3） | AF647-PD-1 (MIH4) | PE-Granzyme-B (QA16A02) | AF647-CD107a (H4A3) |
| - | APC-CD62L  (LT-TD180) | APC-CD49a  (TS2/7) | AF647-Hobit  (Sanquin-Hobit/1) | AF647-TCF1  (7F11A10) | AF647-TNF-α(MAb11) | PE-TIM-3 (7D3) | AF647-perforin(δG9) | BV786-LAG-3(11C3C65) |
| - | - | - | - | - | APC-R700-IL2 (MQ1-17H12) | BV605-CTLA-4 (BNI3) | BV605-Ki-67 | - |
