# Supplementary material for: Identification and validation of an immunogenic subtype of gastric cancer with abundant intratumoural CD103+CD8+ T cells conferring favourable prognosis
Source: Br J Cancer. 2020 Mar 24;122(10):1525–34. doi: 10.1038/s41416-020-0813-y (PMC7217759; doi:10.1038/s41416-020-0813-y)
Supplement: Supplementary file 2 — Supplementary Table S2 [file 41416_2020_813_MOESM2_ESM.docx]

| **Table S2. Association between intratumoral CD103^+^CD8^+^ T cell infiltration with gastric cancer patient characteristics.** | | | | | | | | | | | |
| --- | --- | --- | --- | --- | --- | --- | --- | --- | --- | --- | --- |
| Group | Zhongshan cohort (n=448) | | |  | GSE62254 (n=220) | | |  | GSE84437 (n=431) | | |
|  | CD103^+^CD8^+^ T cells | | |  | CD103^+^CD8^+^ T cell signature | | |  | CD103^+^CD8^+^ T cell signature | | |
|  | high | low | *P*-value |  | high | low | *P*-value |  | high | low | *P*-value |
| Gender  Male  Female | 189  80 | 127  52 | 0.959 |  | 69  28 | 87  36 | 0.933 |  | 214  96 | 82  41 | 0.717 |
| Age  ≥65  ＜65 | 175  94 | 110  69 | 0.499 |  | 51  46 | 67  56 | 0.886 |  | 189  121 | 78  45 | 0.717 |
| Localization  Upper  Middle  Distal | 61  47  161 | 45  20  114 | 0.866 |  | -  -  - | -  -  - | - |  | -  -  - | -  -  - | - |
| Grade  G1  G2  G3 | 15  47  207 | 8  43  128 | 0.423 |  | -  -  - | -  -  - | - |  | -  -  - | -  -  - | - |
| Lauren  Intestinal  Diffuse | 169  100 | 115  64 | 0.837 |  | 51  46 | 69  54 | 0.701 |  | -  - | -  - | - |
| T stage  T1  T2  T3  T4 | 49  37  56  127 | 40  27  29  83 | 0.361 |  | 0  75  21  1 | 0  87  35  1 | 0.313 |  | 9  25  65  211 | 2  13  92  292 | 0.729 |
| N stage  N0  N1  N2  N3 | 102  31  42  94 | 78  20  37  44 | 0.104 |  | 11  53  33  0 | 25  65  33  0 | 0.162 |  | 56  126  105  23 | 24  62  27  10 | 0.103 |
| Adjuvant chemotherapy  With ACT  Without ACT | 111  158 | 80  99 | 0.534 |  | 71  26 | 84  39 | 0.521 |  | -  - | -  - | - |
| Note: ACT= adjuvant chemotherapy; *P*<0.05 marked in bold shows statistical significance. | | | | | | | | | | | |
